# A contrast-enhanced CMR-based risk score integrating microvascular obstruction and intramyocardial hemorrhage extent for prognostic evaluation

**DOI:** 10.1186/s13244-026-02386-2

**Published:** 2026-08-27

**Authors:** Xing-Yu Gu, Ting-Xuan Yin, Ling-Yi Yu, Jin-Yu Zheng, Ming-Lei Li, Yan Zhou, Jun Pu, Jin-Yi Xiang, Jing-Ping Wu, Lian-Ming Wu

**Affiliations:** 1 Department of Radiology, Renji Hospital, School of Medicine, Shanghai Jiao Tong University, Shanghai, China; 2 Department of Cardiology, Renji Hospital, School of Medicine, Shanghai Jiao Tong University, Shanghai, China

**Keywords:** Cardiac magnetic resonance, Myocardial infarction, Major adverse cardiovascular events, Prognosis

## Abstract

**Objectives:**

This study aims to compare the prognostic value of the extent of microvascular obstruction (MVO) combined with intramyocardial hemorrhage (IMH) for major adverse cardiovascular events (MACE) in patients with STEMI.

**Materials and methods:**

We analyzed 568 STEMI patients in this retrospective multicenter study. Microvascular injury was visualized using late gadolinium enhancement for MVO, and T2* mapping and T2-weighted cardiovascular magnetic resonance imaging for IMH. The Youden index was used as the cutoff value to divide the study population into four groups. The Eitel CMR risk score, Glasgow CMR risk score, and GRACE (Global Registry of Acute Coronary Events) score were used as references to form Scores 1 and 2. Score 3 included LVEF ≤ 45%, MVO > 1.06% LV, IMH > 0.47% LV, and the GRACE score.

**Results:**

During a median follow-up of 3.1 years (IQR: 1.7–5.0 years), 108 patients (19%) experienced MACE. Event rates increased stepwise across groups (8.8% to 58.3%). The combination of MVO > 1.06% LV and IMH > 0.47% LV emerged as the strongest predictor of MACE (HR: 9.646, 95% CI: 6.327–14.705 [*p* < 0.001]). Score 3 (integrating GRACE score, LVEF, and quantitative MVO and IMH) demonstrated improved predictive performance for MACE compared to the GRACE score (AUC: 0.748), Score 1 (AUC: 0.825), and Score 2 (AUC: 0.802), achieving an AUC of 0.853.

**Conclusion:**

Our approach integrates quantitative information on MVO and IMH into a risk score that shows better prognostic value than established risk scores.

**Key Points:**

- **Question** Current prognostic tools for ST-elevation myocardial infarction patients lack quantitative combined assessment of microvascular obstruction and intramyocardial hemorrhage, limiting precise risk stratification after reperfusion.
- **Findings** A novel cardiac magnetic resonance score integrating quantitative microvascular obstruction and intramyocardial hemorrhage extent achieved 0.853 area under the curve for major adverse cardiovascular events.
- **Critical Relevance Statement** This study develops a quantitative cardiac magnetic resonance risk score by integrating microvascular obstruction and intramyocardial hemorrhage, offering improved prognostic value for major adverse cardiovascular events in ST-elevation myocardial infarction patients over established clinical scores.

**Graphical Abstract:**

**Figure:**
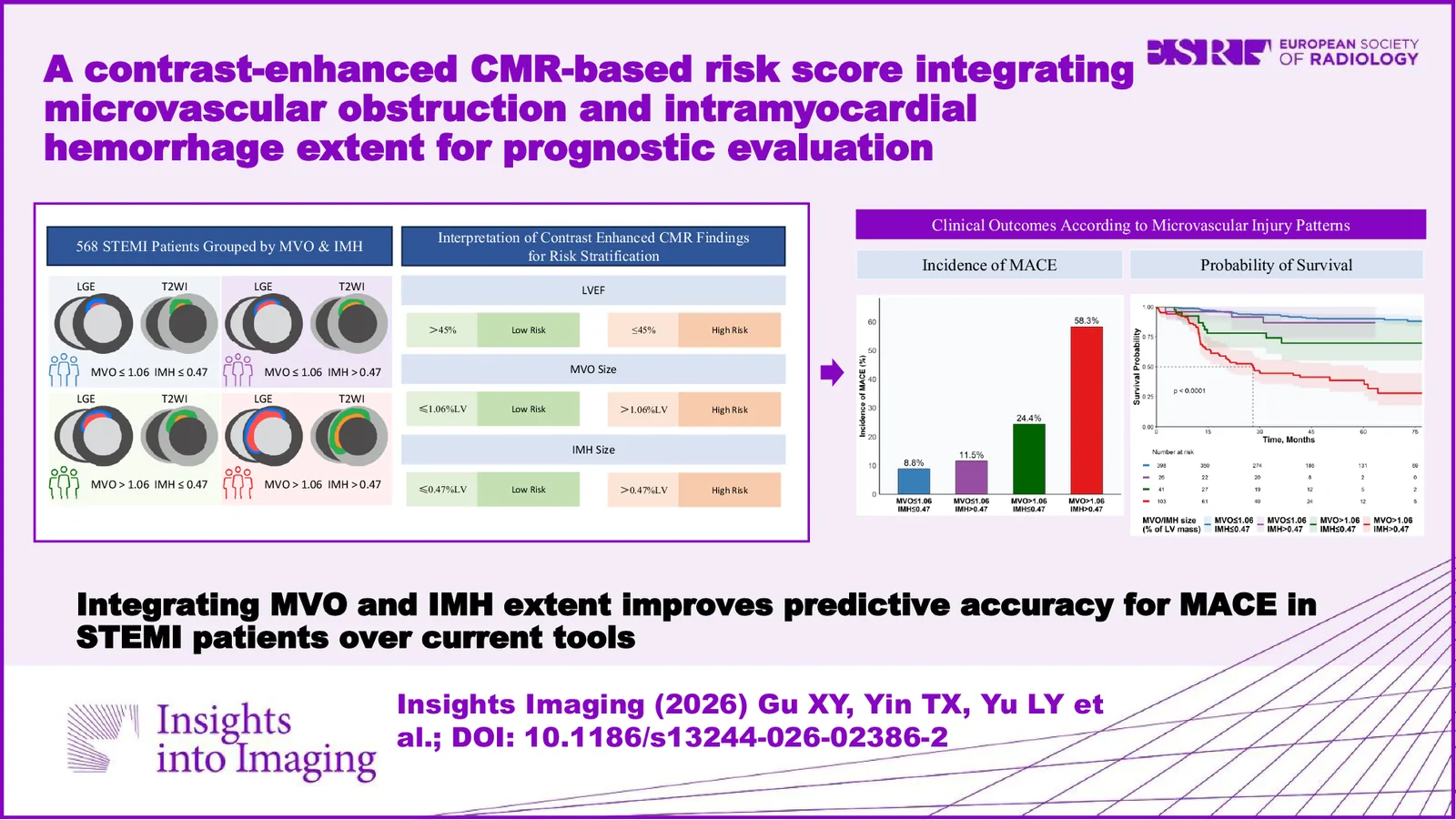

## Introduction

Timely reperfusion strategies, such as percutaneous coronary intervention (PCI), have significantly contributed to the reduction in morbidity and mortality following ST-segment elevation myocardial infarction (STEMI) [1–3]. PCI and coronary revascularization, however, can cause additional injury in the form of microvascular injury. Microvascular obstruction (MVO) [4–6] and intramyocardial hemorrhage (IMH) [7–11], both detectable via cardiovascular magnetic resonance (CMR), are indicative of significant myocardial damage. These lesions restrict the potential for myocardial salvage and are linked to left ventricular dysfunction, which portends an unfavorable prognosis in patients with STEMI. CMR provides the unique ability to visualize myocardial injury through various imaging techniques such as late gadolinium enhancement (LGE), T2-weighted imaging (T2WI), and T2* mapping. These imaging modalities can be used as the gold standard to identify MVO or IMH as hypointense regions within the infarcted myocardium or hyperintense edematous myocardium [12]. Recent studies have evaluated the adverse outcomes in STEMI patients by assessing the presence of MVO and IMH [13], or by quantifying the severity of MVO [5, 14, 15] or IMH [16] individually. However, MVO and IMH represent sequential stages in the progression of microvascular injury [17] and are closely interrelated. Previous studies have not integrated these two factors to quantify their combined impact on the clinical prognosis of patients.

The Global Registry of Acute Coronary Events (GRACE) risk score [18] is a well-established tool for risk stratification in patients with STEMI, predicting both in-hospital and 6-month mortality. However, more specific CMR-based risk scores have also been proposed to enhance prognostic accuracy. Stiermaier et al introduced the Eitel CMR risk score [15], which incorporates left ventricular ejection fraction (LVEF), infarct size, and MVO size to predict a composite endpoint of all-cause death, nonfatal myocardial infarction, and new-onset heart failure at 12 months. Similarly, Bulluck et al developed the Glasgow CMR risk score [19], which includes LVEF and the presence of intramyocardial hemorrhage (IMH) to predict 1- and 5-year all-cause mortality, nonfatal myocardial infarction, and new-onset heart failure. Notably, these models have primarily focused on individual factors of MVO size or IMH presence, rather than a combined assessment of both lesions. While established frameworks like the Canadian Cardiovascular Society (CCS) classification provide a valuable pathophysiological staging of microvascular injury, they still rely on the mere presence or absence of MVO and IMH [20]. This study aims to establish a more granular risk score by combining the extent of MVO and IMH as assessed by CMR, and to compare its prognostic performance with existing risk stratification systems.

## Materials and methods

### Study population and clinical endpoints

This study was approved by the Institutional Review Board of Renji Hospital, Shanghai Jiao Tong University School of Medicine (Approval No.: LY2025-323-B; Date of approval: October 19, 2025).

This retrospective multicenter study included STEMI patients who underwent PCI within 12 h of symptom onset between April 2017 and August 2023. Patients who had a CMR within 7 days (median 4 days, IQR: 3–5 days) of the index event were eligible. The exclusion criteria consisted of: inability to undergo CMR due to claustrophobia, CMR contraindications, or severe renal impairment (GFR < 30 mL/min/1.73 m²); non-ischemic cardiomyopathy; unevaluable T2WI or LGE sequences; documented prior myocardial infarction; and loss to follow-up. The clinical endpoint was a major adverse cardiovascular event (MACE), defined as heart failure, all-cause death, reinfarction, and revascularization. Comprehensive specifications regarding follow-up and outcome adjudication criteria are exhaustively documented in the Supplementary Material. Ethical approval for this study was obtained from the institutional review board, and the study was conducted in accordance with the principles of the Declaration of Helsinki. Written informed consent was waived due to the retrospective study design.

### CMR acquisition and image analysis

Scans were conducted using 3.0-T CMR scanners (Achieva TX, Philips Healthcare; MAGNETOM Skyra, Siemens Healthcare). The imaging protocol included balanced steady-state free precession cine imaging, T2-weighted imaging (T2WI), T2* mapping, and late gadolinium enhancement (LGE) imaging. LGE images were acquired 10 min after the intravenous administration of 0.15 mmol/kg of gadopentetate dimeglumine (Magnevist; Bayer). Additional details regarding the CMR protocol are provided in the Supplementary Table 1.

CMR images were analyzed independently by two radiologists with expertise in CMR (Reader 1: 8 years; Reader 2: 6 years) using CVI42 software (version 6.1.2; Circle Cardiovascular Imaging Inc.). All analyses were performed blinded to clinical outcomes and to each other’s measurements. Endocardial and epicardial contours were semi-automatically traced on short-axis cine images and manually adjusted when necessary. LV functional parameters (LVEF, end-diastolic volume, and end-systolic volume) were automatically calculated by the software and verified by the readers.

**Fig. 1:**
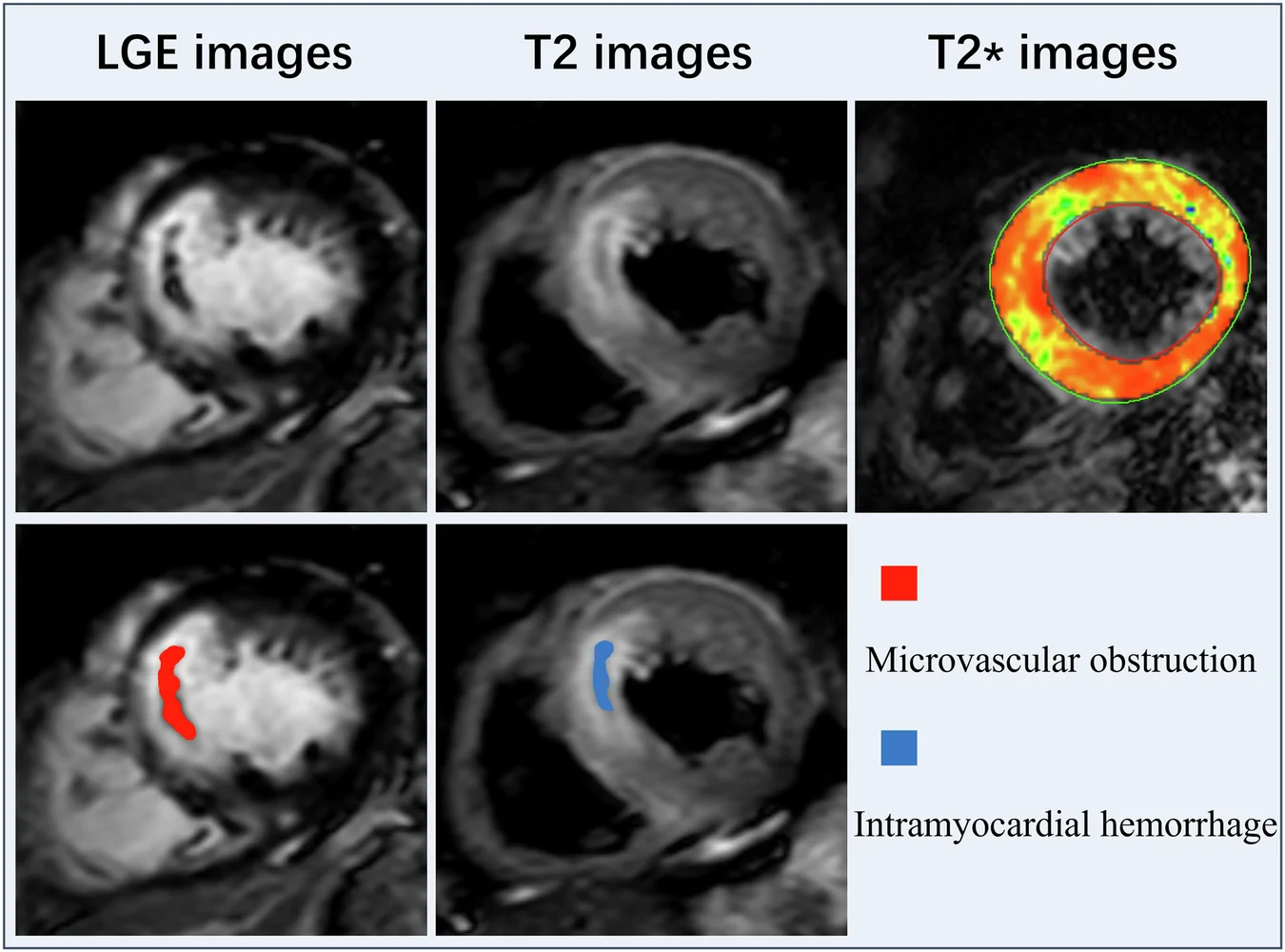
Example of microvascular obstruction and intramyocardial hemorrhage size assessed by cardiac magnetic resonance imaging. LGE, late gadolinium enhancement

The infarct area was defined on LGE images as myocardium with signal intensity > 5 standard deviations (SD) above the mean signal intensity of remote myocardium. MVO was identified on LGE images as hypointense regions (signal void or “dark zones”) within the hyperenhanced infarct area. The presence of MVO was determined by consensus between the two readers before quantification. For MVO quantification, regions of hypoenhancement within the infarct zone on LGE images were manually traced on each short-axis slice and summed. IMH was assessed using a combined approach: (1) initial identification on T2* mapping (three short-axis slices: basal, mid, and apical), defined as regions with T2* values < 20 ms within the infarct zone; (2) patients with IMH presence confirmed by consensus of the two readers were subjected to subsequent quantification on T2WI. IMH was defined as a central hypoenhanced (low-signal) core surrounded by hyperintense edematous myocardium (signal intensity > 2 SD above remote myocardium). MVO and IMH were manually delineated and quantified in LGE images and T2WI, respectively (Fig. 1). The above quantification is expressed as a percentage of left ventricular myocardial mass (LVMM). Quantitative measurements were averaged between the two readers after confirmation of excellent interobserver reproducibility.

### Clinical risk score and CMR risk score

The GRACE score [18]: we used GRACE 2.0 [21], including age, heart rate, systolic blood pressure on admission, creatinine, cardiac arrest on admission, ST-segment deviation on electrocardiogram, abnormal cardiac enzymes, and Killip class (https://www.mdcalc.com/grace-acs-risk-mortality-calculator).

The Eitel CMR risk score [15]: we assign 1 point for LVEF ≤ 47% or infarct size ≥ 19% of LV mass, and 2 points for MVO ≥ 1.4% of LV mass. Patients with STEMI could be stratified into low risk (score: 0 or 1) and high risk (score: > 1).

The Glasgow CMR risk score [19]: patients with STEMI could be stratified into low risk (LVEF > 45% and no IMH), intermediate risk (LVEF > 45% and IMH), and high risk (LVEF ≤ 45%).

### The CCS classification

The Canadian Cardiovascular Society (CCS) categorizes myocardial infarction into four stages of increasing severity of myocardial injury [20]. Stage 1: myocardial edema only; Stage 2: evidence of necrosis without microvascular injury; Stage 3: myocardial infarction (MI) with MVO; and Stage 4: MI with MVO and IMH.

### Statistical analysis

Continuous variables were assessed for normality using the Shapiro–Wilk test. Normally distributed variables are presented as mean ± standard deviation (SD): between two groups, they were compared using unpaired Student’s *t*-tests, while among four groups, they were analyzed by one-way ANOVA with Bonferroni post hoc correction. Non-normally distributed variables are expressed as median (interquartile range) and compared using Mann–Whitney U tests for two-group comparisons or Kruskal–Wallis tests with Dunn’s post hoc adjustment for multi-group analyses. Categorical variables are presented as counts (%) and analyzed by chi-square or Fisher’s exact tests as appropriate.

The Youden index was used to determine the optimal cutoff values (MVO > 1.06% LV and IMH > 0.47% LV) for dividing the study population into four groups: (1) MVO ≤ 1.06% LV & IMH ≤ 0.47% LV; (2) MVO ≤ 1.06% LV & IMH > 0.47% LV; (3) MVO > 1.06% LV & IMH ≤ 0.47% LV; and (4) MVO > 1.06% LV & IMH > 0.47% LV. To identify predictors of MACE, we performed univariable and multivariable Cox regression analyses. Significant risk factors from univariate analyses (*p* < 0.05) and basic factors are entered simultaneously into the multivariable model (backward stepwise regression method). Hazard ratios (HR) were calculated with a 95% confidence interval (95% CI). We compared the cumulative MACE incidence in each group using Kaplan–Meier survival curves.

For prognostic assessment, three risk prediction scores were constructed and compared with the ROC:

- Score 1 incorporated the GRACE score and the Eitel CMR risk score parameters (LVEF ≤ 47%, infarct size ≥ 19% LV, MVO > 1.4% LV).
- Score 2 incorporated the GRACE score and the Glasgow CMR risk score parameters (LVEF ≤ 45%, presence of IMH).
- Score 3, our proposed score, incorporated the GRACE score, LVEF ≤ 45%, and quantitative MVO and IMH extent. The specific cutoffs were derived from the study data, as detailed in the “Results.”

Receiver-operating characteristic (ROC) curves were constructed using the predicted probabilities generated for each score. The area under the receiver-operating characteristic curve (AUC), net reclassification improvement (NRI), and integrated discrimination improvement (IDI) were used to compare different parameters for predicting MACE, and *p* < 0.05 was considered statistically significant.

The intra- and interobserver reproducibility of cardiac magnetic resonance (CMR) parameters was assessed using the intraclass correlation coefficient (ICC). For intra-observer reproducibility, a single observer repeated measurements under identical conditions, while interobserver reproducibility was evaluated between two independent observers. Results are presented as ICC values with 95% confidence intervals (95% CI).

Model performance was internally validated using bootstrap resampling (B = 1000) to correct for overfitting.

Statistical analysis was performed using SPSS version 25 (IBM Corporation) and R (version 4.3.2; R Foundation).

## Results

### Patient characteristics and CMR characteristics

**Fig. 2:**
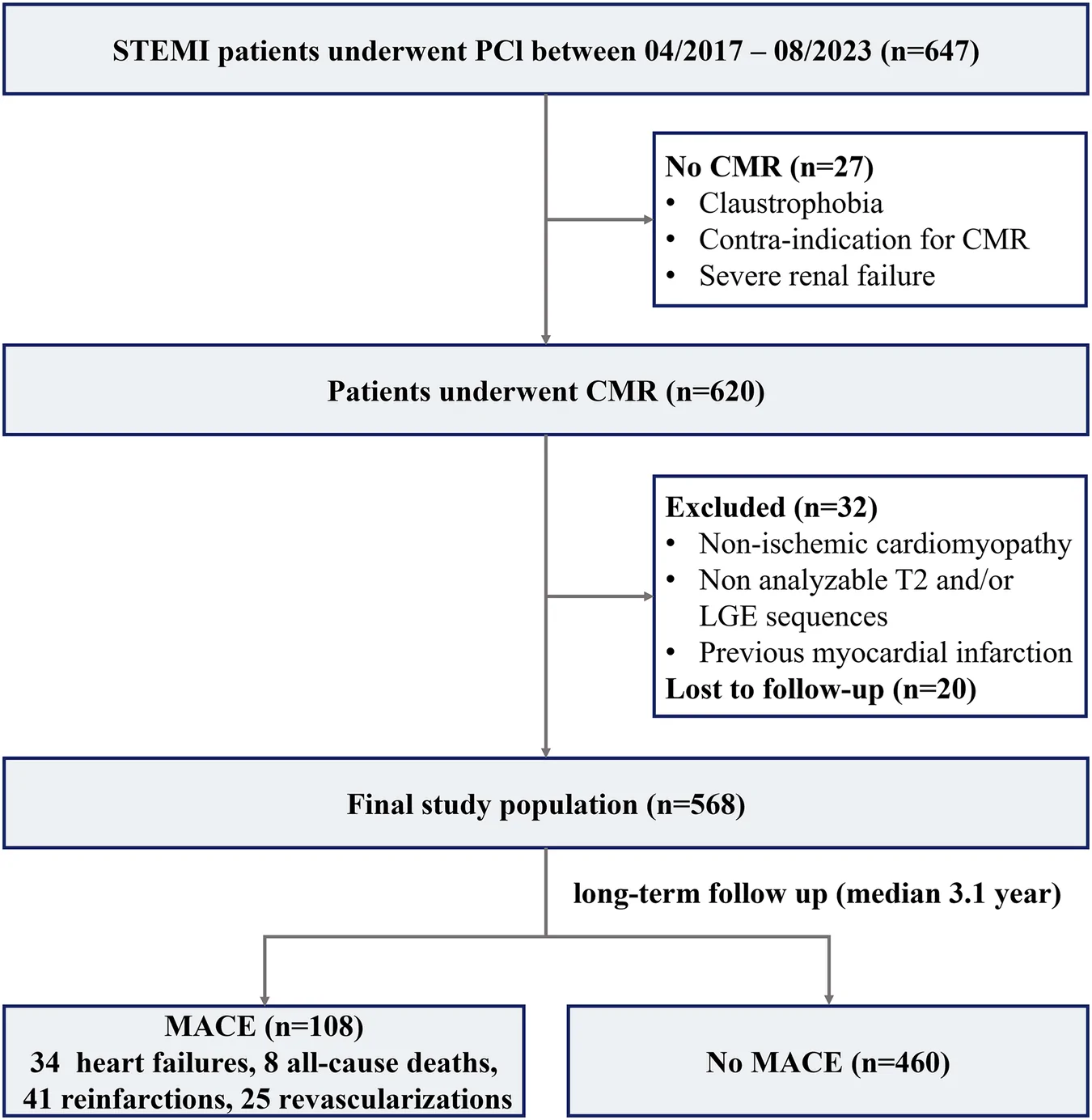
Study flowchart. STEMI, ST-elevation myocardial infarction; PCI, percutaneous coronary intervention; CMR, cardiac magnetic resonance; LGE, late gadolinium enhancement; MACE, major adverse cardiovascular events

A total of 568 eligible STEMI patients (82% male) were included in this pooled individual patient analysis at a median age of 61 years (Q1-Q3: 53–67 years). Patients were divided into two groups based on the occurrence of MACE events (Fig. 2), and the baseline characteristics of the study population are shown in Table 1. In the group that developed MACE, the incidence of Killip class > 1 (*p* < 0.001) and GRACE scores (median: MACE 110 vs. No MACE 85; *p* < 0.001) were higher. Significant differences were also observed in peak brain natriuretic peptide levels (*p* = 0.020) and total cholesterol levels (*p* = 0.033). Meanwhile, age, hypertension, and angiographic characteristics presented no group differences (all *p* > 0.05).

**Table 1:** SEPLA between MACE and non-MACE among STEMI patients

	All patients (*n* = 568)	MACE (*n* = 108)	No MACE (*n* = 460)	*p*-value
Age, years	61 (53–67)	62 (55–69)	61 (52–67)	0.164
Men, *n* (%)	467 (82)	90 (83)	377 (82)	0.736
BSA, m^2^	1.8 (1.7–1.9)	1.8 (1.7–1.9)	1.8 (1.7–1.9)	0.355
BMI, kg/m^2^	24.7 (22.7–27.0)	24.2 (22.7–26.6)	24.8 (22.7–27.0)	0.323
Heart rate, bpm	75 (68–83)	76 (68–82)	74 (68–83)	0.477
GRACE score	88 (75–109)	110 (92–127)	85 (72–102)	**< 0.001**
Risk factors, *n* (%)				
Hypertension	274 (48)	55 (51)	219 (48)	0.535
Diabetes mellitus	219 (39)	50 (46)	169 (37)	0.066
Dyslipidemia	146 (26)	21 (19)	125 (27)	0.098
Smoking history	265 (47)	56 (52)	209 (45)	0.229
Killip class				**< 0.001**
I	461 (81)	73 (68)	388 (84)	
II	85 (15)	24 (22)	61 (13)	
III	13 (2)	7 (6)	6 (1)	
IV	9 (2)	4 (4)	5 (1)	
Laboratory indices				
TnI_max_, ng/mL	11.7 (3.0–24.9)	13.0 (2.9–23.2)	11.0 (3.0–24.9)	0.699
BNP_max_, pg/mL	146 (57–365)	233 (57–537)	127 (57–332)	**0.020**
NT-proBNP, pg/mL	939 (528–1469)	997 (575–1588)	931 (525–1433)	0.150
CRP_max_, mg/L	3.1 (0.7–20.8)	3.5 (0.6–19.3)	2.9 (0.7–21.9)	0.899
hsCRP, mg/L	4.8 (1.6–11.2)	4.5 (1.3–11.2)	4.8 (1.6–11.2)	0.669
ApoA, mg/dL	98 (49–174)	126 (46–192)	94 (50–171)	0.174
TC, mg/dL	4.5 (3.7–5.4)	4.2 (3.6–5.2)	4.5 (3.7–5.4)	**0.033**
TG, mg/dL	1.5 (1.0–2.3)	1.5 (1.0–2.3)	1.5 (1.0–2.4)	0.518
LDL, mmol/L	2.7 (2.2–3.4)	2.7 (2.0–3.3)	2.7 (2.2–3.5)	0.179
HDL, mmol/L	1.0 (0.9–1.2)	1.0 (0.8–1.3)	1.0 (0.9–1.2)	0.657
Angiographic				
Infarct artery				0.642
LAD	310 (55)	56 (52)	254 (55)	
LCX	64 (11)	11 (10)	53 (12)	
RCA	194 (34)	41 (38)	153 (33)	
Number of diseased vessels				0.072
1	433 (76)	81 (75)	352 (77)	
2	85 (15)	22 (20)	63 (14)	
3	50 (9)	5 (5)	45 (10)	
TIMI flow grade before PCI				0.285
0	387 (68)	81 (75)	306 (67)	
1	24 (4)	5 (5)	19 (4)	
2	42 (7)	7 (6)	35 (8)	
3	115 (20)	15 (14)	100 (22)	
TIMI flow grade after PCI				0.170
0	14 (2)	3 (3)	11 (2)	
1	2 (0)	0 (0)	2 (0)	
2	3 (1)	2 (2)	1 (0)	
3	548 (97)	102 (95)	446 (97)	
Medications, *n* (%)				
Aspirin	351 (62)	69 (64)	282 (61)	0.542
Other oral antiplatelet agents	351 (62)	69 (64)	282 (61)	0.542
Statin	347 (61)	73 (68)	274 (60)	0.098
ACEI/ARB	203 (36)	35 (33)	168 (37)	0.459
β-Blockers	306 (54)	60 (56)	246 (53)	0.627
Diuretic	78 (14)	21 (20)	57 (12)	0.050
Insulin/oral hypoglycemic agent	80 (14)	17 (16)	63 (14)	0.557

Data are presented as median (interquartile range) or mean ± standard deviation or *n*/*N* (%). Values in bold indicate a *p*-value < 0.05

*MACE* major adverse cardiovascular events, *STEMI* ST-segment elevation myocardial infarction, *BSA* body surface area, *BMI* body mass index, *GRACE* Global Registry of Acute Coronary Events, *cTnI*_*max*_ peak troponin I, *BNP*_*max*_ peak brain natriuretic peptide, *CRP*_*max*_ peak C-reactive protein, *TC* total cholesterol, *TG* triglycerides, *LDL* low-density lipoprotein, *HDL* high-density lipoprotein, *LAD* left anterior descending, *LCX* left circumflex, *RCA* right coronary artery, *TIMI* thrombolysis in myocardial infarction, *ACEI* angiotensin converting enzyme inhibitor, *ARB* angiotonin receptor blocker

### CMR characteristics

The CMR characteristics are summarized in Table 2. 269 (47%) patients developed MVO and 195 (34%) patients developed IMH, with higher rates of MVO (71% vs. 42%) and IMH (65% vs. 27%) in the MACE cohort than in the no MACE cohort. Patients experiencing MACE demonstrated smaller LVEF (47% vs. 49%, *p* = 0.004) and larger LV mass index (70.5% vs. 60.5%, *p* < 0.001), MVO size (1.82% vs. 0%, *p* < 0.001), and IMH size (0.82% vs. 0%, *p* < 0.001). The optimal cutoff values for predicting MACE, as determined by the Youden index, were identified as MVO of 1.06% of left ventricular (LV) mass and IMH of 0.47% of LV mass. Based on these two indicators, all patients were divided into four groups: MVO ≤ 1.06% LV & IMH ≤ 0.47% LV (*n* = 398, 70.1%), MVO ≤ 1.06% LV & IMH > 0.47% LV (*n* = 26, 4.6%), MVO > 1.06% LV & IMH ≤ 0.47% LV (*n* = 41, 7.2%), and MVO > 1.06% LV & IMH > 0.47% LV (*n* = 103, 18.1%). Cardiac function deteriorated, and the incidence of MACE increased (9% to 58%, *p* < 0.001) with the stepwise increasing severity of microvascular injury (Table 3). The LVEF exhibited a progressive decline across groups (50% vs. 47% vs. 46% vs. 43%, *p* < 0.001). Infarct size exhibited significant differences across the four groups, with the largest extent of late gadolinium enhancement observed in MVO > 1.06% LV & IMH > 0.47% LV (median 30%, IQR: 22–39%) compared to MVO ≤ 1.06% LV & IMH ≤ 0.47% LV (median 17%, IQR: 10–27%; *p* < 0.001). Clinical and imaging parameters according to the CMR phenotypes (MVO−/IMH−, MVO+/IMH−, and IMH+) are provided in Supplementary Table 2 and Supplementary Table 3. Differences stratified by MVO cutoff value and by IMH cutoff value are presented in Supplementary Table 4, respectively. Supplementary Table 5 integrates CCS stages with quantitative injury groups. Even within CCS-4 (definite MI with MVO and IMH), MACE ranged from 3.6% when both injuries were small to 58.3% when both were large. Conversely, when microvascular injury was limited (MVO ≤ 1.06% LV and IMH ≤ 0.47% LV), event rates were low and similar across CCS-2–4 (10.4%, 4.7%, 3.6%). Thus, quantitative assessment refines the broad prognostic heterogeneity captured by qualitative CCS-4.

**Table 2:** CMR parameters between MACE and non-MACE among STEMI patients

Parameters	All patients (*n* = 568)	MACE (*n* = 108)	No MACE (*n* = 460)	*p*-value
LV EDVI (mL/m^2^)	72.5 (60.3–86.1)	79.6 (67.0–92.0)	71.8 (59.5–84.6)	**< 0.001**
LV ESVI (mL/m^2^)	34.0 (26.0–45.4)	40.2 (31.9–54.5)	33.6 (25.0–44.0)	**< 0.001**
LVEF (%)	49 (39–56)	47 (37–52)	49 (40–57)	**0.004**
LV SVi (mL/m^2^)	36.1 (27.8–43.4)	36.3 (30.8–44.1)	36.1 (27.3–43)	0.238
LV mass index (g/m^2^)	61.1 (50.5–75.4)	70.5 (55.9–80.2)	60.5 (49.7–73.8)	**0.001**
LV aneurysm, *n* (%)	96 (17)	19 (18)	77 (17)	0.831
LV thrombus, *n* (%)	48 (8)	11 (10)	37 (8)	0.471
LGE, % of LV	20 (12–31)	26 (18–37)	20 (11–29)	**< 0.001**
MVO present, *n* (%)	269 (47)	77 (71)	192 (42)	**< 0.001**
MVO, % of LV mass	0 (0–1.08)	1.82 (0–4.03)	0 (0–0.68)	**< 0.001**
IMH present, *n* (%)	195 (34)	70 (65)	125 (27)	**< 0.001**
IMH, % of LV mass	0 (0–0.42)	0.82 (0–2.76)	0 (0–0.17)	**< 0.001**

Data are presented as median (interquartile range) or mean ± standard deviation or *n*/*N* (%). Values in bold indicate a *p*-value < 0.05

*MACE* major adverse cardiovascular events, *STEMI* ST-segment elevation myocardial infarction, *LV* left ventricle, *EDVI* end-diastolic volume index, *ESVI* end-systolic volume index, *LVEF* left ventricular ejection fraction, *SVi* stroke volume index, *LGE* late gadolinium enhancement, *MVO* microvascular obstruction, *IMH* intramyocardial hemorrhage

**Table 3:** The distribution differences of CMR parameters categorized by the MVO and IMH cutoff value

	MVO ≤ 1.06 IMH ≤ 0.47 (*n* = 398)	MVO ≤ 1.06 IMH > 0.47 (*n* = 26)	MVO > 1.06 IMH ≤ 0.47 (*n* = 41)	MVO > 1.06 IMH > 0.47 (*n* = 103)	*p*-value
MACE, *n* (%)	35 (9)	3 (12)	10 (24)	60 (58)	**< 0.001**
Survival time	1277 (765–2037)	1208 (862–1401)	766 (373–1463)	620 (364–1250)	**< 0.001**
LV EDVI (mL/m^2^)	70.7 (58.2–83.6)	74.7 (68.9–89.4)	79.6 (72.3–95.4)	79.0 (67.0–91.4)	**< 0.001**
LV ESVI (mL/m^2^)	32.0 (23.6–42.8)	39.4 (31.4–45.3)	43.0 (31.7–57.1)	40.8 (33.0–56.6)	**< 0.001**
LVEF (%)	50 (42–57)	47 (41–51)	46 (30–51)	43 (37–50)	**< 0.001**
LV SVi (mL/m^2^)	36.1 (27.4–43.4)	38.5 (33.2–46.8)	37.0 (32.1–44.2)	34.1 (27.7–42.3)	0.296
LV mass index (g/m^2^)	60.5 (48.7–75.4)	63.9 (58.2–75.9)	63.1 (54.3–75.5)	65.7 (54.8–75.8)	**0.042**
LV aneurysm, *n* (%)	56 (14)	6 (23)	12 (29)	22 (21)	**0.031**
LV thrombus, *n* (%)	26 (7)	6 (23)	5 (12)	11 (11)	**0.015**
LGE, % of LV	17 (10–27)	28 (24–32)	23 (20–33)	30 (22–39)	**< 0.001**

Data are presented as median (interquartile range) or mean ± standard deviation or *n*/*N* (%). Values in bold indicate a *p*-value < 0.05. The cutoff values for MVO and IMH are expressed as a percentage of left ventricular mass

*CMR* cardiac magnetic resonance, *MVO* microvascular obstruction, *IMH* intramyocardial hemorrhage, *MACE* major adverse cardiovascular events, *LV* left ventricle, *EDVI* end-diastolic volume index, *ESVI* end-systolic volume index, *LVEF* left ventricular ejection fraction, *SVi* stroke volume index, *LGE* late gadolinium enhancement

### Clinical outcomes according to the extent of microvascular injury

Over a median follow-up period of 3.1 years, MACE was observed in 108 patients, comprising 34 cases of heart failure, 8 all-cause deaths, 41 recurrent myocardial infarctions, and 25 revascularization procedures. In the univariable Cox regression analysis, Killip class (HR: 1.736; 95% CI: 1.377–2.189; *p* < 0.001), LVEF (HR: 0.978; 95% CI: 0.964–0.991; *p* = 0.001), and microvascular injury subgroups were significantly associated with the primary MACE endpoint. After adjustment for clinical and CMR parameters of these variables, Group 4 (MVO > 1.06% LV and IMH > 0.47% LV) demonstrated the most pronounced association with an elevated hazard ratio (HR: 9.646, 95% CI: 6.327–14.705; *p* < 0.001). In contrast, Group 2 (MVO ≤ 1.06% LV and IMH > 0.47% LV) did not achieve statistical significance in the multivariable analysis (HR: 1.317, 95% CI: 0.403–4.303; *p* = 0.649) (Table 4).

**Table 4:** Cox regression analysis for predicting MACE among STEMI patients

	Univariate analysis		Multivariate analysis	
	HR (95% CI)	*p*-value	HR (95% CI)	*p*-value
Age, years	1.012 (0.994–1.030)	0.185		
Men, *n* (%)	1.153 (0.695–1.913)	0.582		
Heart rate, bpm	1.007 (0.993–1.022)	0.309		
Killip class	1.736 (1.377–2.189)	**< 0.001**		
LV EDVI (mL/m^2^)	1.011 (1.005–1.017)	**< 0.001**		
LV ESVI (mL/m^2^)	1.011 (1.005–1.018)	**0.001**		
LVEF (%)	0.978 (0.964–0.991)	**0.001**		
LV mass index (g/m^2^)	1.012 (1.005–1.020)	**0.001**	1.012 (1.004–1.020)	**0.003**
LGE, % of LV	1.026 (1.015–1.036)	**< 0.001**		
Grouped by MVO and IMH				
Group 1	Reference		Reference	
Group 2	1.499 (0.460–4.888)	0.502	1.317 (0.403–4.303)	0.649
Group 3	3.772 (1.864–7.631)	**< 0.001**	3.761 (1.857–7.618)	**< 0.001**
Group 4	9.835 (6.456–14.984)	**< 0.001**	9.646 (6.327–14.705)	**< 0.001**

Values in bold indicate a *p*-value < 0.05. Multivariate COX analysis was conducted using the backward stepwise regression method

Grouped by MVO and IMH: Group 1: MVO ≤ 1.06% LV & IMH ≤ 0.47% LV, Group 2: MVO ≤ 1.06% LV & IMH > 0.47% LV, Group 3: MVO > 1.06% LV & IMH ≤ 0.47% LV, Group 4: MVO > 1.06% LV & IMH > 0.47% LV

*STEMI* ST-segment elevation myocardial infarction, *CI* confidence interval, *LV* left ventricle, *EDVI* end-diastolic volume index, *ESVI* end-systolic volume index, *LVEF* left ventricular ejection fraction, *LGE* late gadolinium enhancement, *MVO* microvascular obstruction, *IMH* intramyocardial hemorrhage

**Fig. 3:**
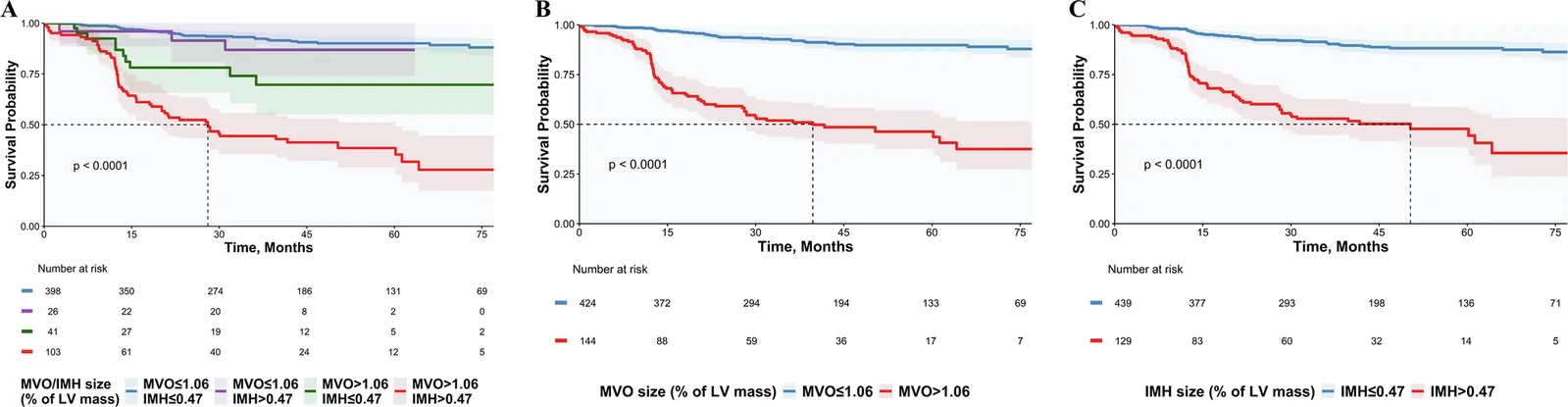
Kaplan–Meier survival analysis for major adverse cardiovascular events (MACE) based on microvascular injury extent. Patients were stratified by cardiovascular magnetic resonance (CMR)-derived quantitative parameters. **A** Survival curves for patients stratified by the combined status of microvascular obstruction (MVO) and intramyocardial hemorrhage (IMH). **B** Survival curves for patients stratified by MVO size alone. **C** Survival curves for patients stratified by IMH size alone. In all panels, the cutoff values are expressed as a percentage of left ventricular (LV) mass

The Kaplan–Meier estimates for the primary MACE endpoints showed a stepwise increase in adverse events across groups. Patients with MVO > 1.06% LV & IMH ≤ 0.47% LV were at a significantly increased risk of adverse events compared to both MVO ≤ 1.06% LV & IMH ≤ 0.47% LV and MVO ≤ 1.06% LV & IMH > 0.47% LV groups, whereas the highest rate of events was observed in MVO > 1.06% LV & IMH > 0.47% LV (Fig. 3). The Kaplan–Meier curves grouped by MVO cutoff and IMH cutoff are shown in Fig. 3, respectively.

### Prognostic performance of the CMR risk scores

**Fig. 4:**
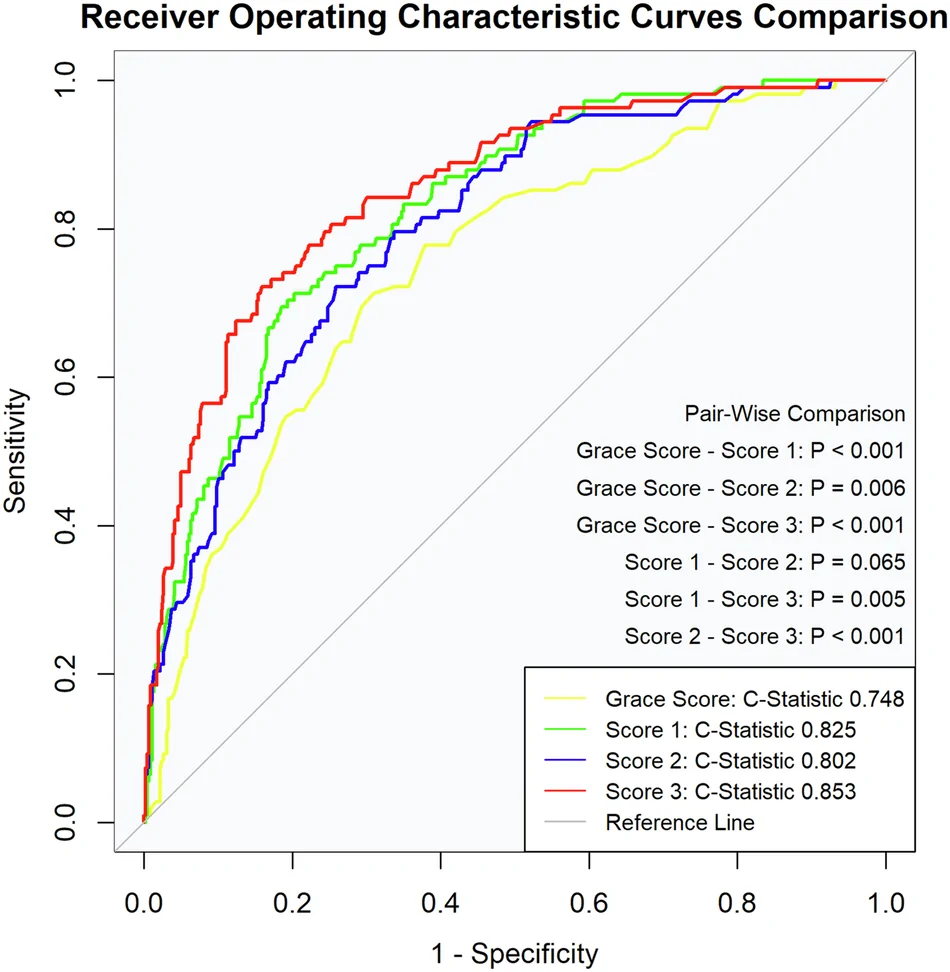
ROC curve comparisons of the 3 different scores against the GRACE score to predict MACE. Score 1 (GRACE score, LVEF ≤ 47%, MI Size ≥ 19% LV and MVO > 1.4% LV). Score 2 (GRACE score, LVEF ≤ 45% and IMH). Score 3 (GRACE score, LVEF ≤ 45%, MVO > 1.06% LV and IMH > 0.47% LV). ROC, receiver-operating characteristic; GRACE, Global Registry of Acute Coronary Events; MACE, major adverse cardiovascular events; LVEF, left ventricular ejection fraction; MI, myocardial infarction; LV, left ventricle; MVO, microvascular obstruction; IMH, intramyocardial hemorrhage

The area under the receiver-operating characteristic curve (AUC, equivalent to C-statistic) for the GRACE score in predicting the composite outcome was 0.748. When the GRACE score was combined with the Eitel CMR risk score parameters (LVEF ≤ 47%, MI size ≥ 19% LV, and MVO > 1.4% LV) to form Score 1, the AUC improved to 0.825. Similarly, the integration of the GRACE score with the Glasgow CMR risk score parameters (LVEF ≤ 45% and presence of IMH) to form Score 2 resulted in an AUC of 0.802. Notably, the incorporation of quantitative values of MVO and IMH into Score 3 (GRACE score, LVEF ≤ 45%, MVO > 1.06% LV, and IMH > 0.47% LV) significantly enhanced the AUC to 0.853. Comparison of the AUC revealed statistically significant differences between Score 3 and the other scores. Specifically, Score 3 demonstrated a significantly higher AUC compared to Score 1 (*p* = 0.005) and Score 2 (*p* < 0.001). While no significant difference was found between Score 1 and 2 (*p* = 0.065). All three composite scores exhibited improved performance compared to the GRACE score alone (GRACE score vs. score 1: *p* < 0.001; GRACE score vs. score 2: *p* = 0.006; GRACE score vs. score 3: *p* < 0.001) (Fig. 4). Score 3 demonstrated enhanced risk stratification compared to the GRACE score alone (Table 5), with an NRI of 35.35% (95% CI: 24.92–45.79%, *p* < 0.001) and an IDI of 19.10% (95% CI: 14.65–23.55%, *p* < 0.001). Supplementary Table 6 presents the AUC for specific MACE outcomes across each model, with Score 3 demonstrating the highest predictive efficacy for all specific outcomes. The AUC for Score 3 are as follows: heart failure, 0.843; all-cause death, 0.890; reinfarction, 0.850; and revascularization, 0.847. The improved performance of Score 3 was maintained after bootstrap-based optimism correction (corrected AUC = 0.849; Supplementary Table 7). Furthermore, the optimism estimates for all scores were small, ranging from −0.001 to 0.006.

**Table 5:** Models associated with CMR parameters for improving the predictive ability of MACE among acute myocardial infarction patients

Reference models	Models	NRI		IDI	
		Index (95% CI)	*p*-value	Index (95% CI)	*p*-value
Grace score	Score 1	20.32% (10.58–30.06%)	**< 0.001**	11.81% (8.21–15.42%)	**< 0.001**
	Score 2	18.14% (8.84–27.44%)	**< 0.001**	8.52% (5.64–11.40%)	**< 0.001**
	Score 3	35.35% (24.92–45.79%)	**< 0.001**	19.10% (14.65–23.55%)	**< 0.001**
Score 1	Score 2	−2.18% (−9.26 to 4.90%)	0.546	−3.29% (−5.93 to −0.65%)	**0.015**
	Score 3	15.03% (7.67–22.39%)	**< 0.001**	7.29% (4.74–9.84%)	**< 0.001**
Score 2	Score 3	17.21% (8.76–25.67%)	**< 0.001**	10.58% (8.01–13.14%)	**< 0.001**

Score 1 (GRACE score, LVEF ≤ 47%, MI Size ≥ 19% LV and MVO > 1.4% LV)

Score 2 (GRACE score, LVEF ≤ 45% and IMH)

Score 3 (GRACE score, LVEF ≤ 45%, MVO > 1.06% LV and IMH > 0.47% LV)

*CMR* cardiac magnetic resonance, *MACE* major adverse cardiovascular events, *NRI* net reclassification improvement, *IDI* integrated discrimination improvement, *CI* confidence interval, *GRACE* Global Registry of Acute Coronary Events, *LVEF* left ventricular ejection fraction, *MI* myocardial infarction, *LV* left ventricle, *MVO* microvascular obstruction, *IMH* intramyocardial hemorrhage

Values in bold indicate a p-value < 0.05

### Intra- and interobserver variability

Intraclass correlation coefficients (ICCs) for CMR parameters demonstrated excellent reproducibility, with all values exceeding 0.90 (Supplementary Table 8).

## Discussion

This is the first multicenter study to integrate the extent of MVO and IMH in STEMI patients treated with primary PCI to assess their association with MACE. The major findings of this study are summarized as follows: (1) Among patients with differing degrees of microvascular injury, infarct size and short-term LV function parameters exhibited significant variation. (2) The classification based on the degree of MVO and IMH was independently associated with MACE. Patients with more severe microvascular injury exhibited worse survival and higher event rates. MVO > 1.06% LV and IMH > 0.47% LV emerged as the strongest predictors of MACE. (3) The score integrating MVO and IMH quantification values presented in this study exhibited enhanced prognostic performance compared to the established Eitel and Glasgow CMR risk scores. Our findings highlight the essential role of incorporating the extent of both MVO and IMH in the risk stratification of STEMI patients. This study improves the prognostic value of clinical outcomes assessed by CMR imaging and offers significant insights for guiding subsequent patient management and treatment.

### Comprehensive quantification of dual phases of microvascular obstruction

Persistent microvascular dysfunction in reperfused STEMI patients can be accurately assessed by CMR imaging [12, 22]. MVO and IMH serve as significant prognostic tissue biomarkers of microvascular injury, negatively impacting cardiovascular prognosis [17, 23, 24]. De Waha et al [5, 14, 15] utilized the presence and degree of MVO to stratify patient prognosis. Klem et al [16, 25] employed the quantification of IMH as a clinical predictor. These methods overlook the continuum of microvascular injury progression. Analysis of a single stage alone does not provide a comprehensive assessment of the severity of microvascular injury after PCI in STEMI patients. Lechner et al [13], while innovatively combining the presence of MVO and IMH to assess patient prognosis, did not quantify the extent of microvascular injury in their study. Our findings extend prior research and emphasize the importance of combining these two quantitative metrics and subgrouping based on the Youden index. In this study, MVO was quantified by LGE [12, 26, 27], while IMH was identified by T2* mapping [12, 28, 29] and measured on T2WI [12, 30–32]. Patients in the subgroup with MVO > 1.06% LV & IMH > 0.47% LV exhibited the worst clinical outcomes and represented the most severe microvascular injury. After adjusting for clinical and CMR parameters, the difference in clinical outcome between the two groups of patients with MVO ≤ 1.06% LV was not significant, whereas the risk of MACE in patients progressively increased with higher levels of microvascular injury. Therefore, the prognostic status of STEMI patients is determined by the combined severity of MVO and IMH.

### Refinement of the existing classification and scoring system

CCS delineates four stages of microvascular injury as edema, necrosis, MVO only, and MVO with IMH [20]. Recent studies [5, 13] have achieved groundbreaking results in risk stratification based on this classification. In our study, the latter two stages were further stratified by quantifying the size of MVO and IMH, yielding more specific stratification indices to enhance risk prediction.

The GRACE score [33], introduced in 2003, is a simple and reliable tool for predicting mortality in acute coronary syndrome (ACS). It has been validated across diverse cohorts for predicting mortality [18, 34, 35]. Recently, it was optimized as the simplified GRACE 2.0 algorithm [21].With the advancement of CMR imaging technology, the assessment of microvascular injury in STEMI patients after reperfusion therapy has become more detailed. Recent studies have introduced the Eitel CMR risk score [15], which includes the degree of MVO, and the Glasgow CMR risk score [19], which includes the presence of IMH. These scores have significantly increased the accuracy of risk prediction by incorporating CMR-derived metrics into clinical risk stratification. By quantitatively grading MVO and IMH, we have extended the existing risk prediction model to highlight the relationship between varying degrees of MVO combined with IMH and MACE events such as heart failure, all-cause death, reinfarction, and revascularization. This optimized risk prediction not only further improves the predictive efficacy of CMR but also provides important support for clinical decision-making and helps optimize treatment strategies for individualized management of patients.

### Future directions

Future studies should prioritize the external validation of Score 3, specifically targeting the generalizability of its MVO and IMH cutoff values. Furthermore, recent studies [36, 37] have shown that myocardial strain measured by CMR can be used to assess overall myocardial function in patients with STEMI and to provide new imaging biomarkers for predicting cardiac recovery. These biomarkers have the potential to be incorporated into predictive models to further refine stratification metrics in the future.

### Limitations

This investigation is also subject to certain limitations. First, the retrospective nature of our study may introduce potential selection bias and unmeasured confounders. Second, our study cohort comprised patients without contraindications to CMR and with complete clinical data for GRACE score calculation; hence, our findings may not be generalizable to all STEMI patients. Third, temporal differences in CMR image acquisition and variability in image quality across multiple centers may influence diagnostic accuracy. Fourth, although our study benefited from a multicenter design, this still constitutes an internal validation; therefore, external validation in independent cohorts is necessary to confirm the generalizability of our findings and the proposed Score 3. Lastly, the T2* and T2WI sequences used in this study were limited to three-layer imaging, which may result in the underestimation of overall IMH measurements due to missed regions.

## Conclusions

Our study integrates the extent of MVO and IMH to grade patients and incorporates quantitative data into a new risk score, thereby refining prior incomplete assessments of microvascular injury stages. This score demonstrated improved prognostic value for clinical outcomes, including MACE, in STEMI patients compared to existing risk scores, thus offering a more robust CMR-based tool for clinical management. Future studies are warranted to validate our findings and further refine the scoring system by incorporating additional metrics, such as myocardial strain.

## Supplementary information


ELECTRONIC SUPPLEMENTARY MATERIAL


## Data Availability

The data that support the findings of this study are available from the corresponding author on reasonable request.

## Abbreviations

AUC: Area under the curve
CMR: Cardiac magnetic resonance
GRACE: Global Registry of Acute Coronary Events
IDI: Integrated discrimination improvement
IMH: Intramyocardial hemorrhage
LGE: Late gadolinium enhancement
LV: Left ventricle
LVEF: Left ventricular ejection fraction
MACE: Major adverse cardiovascular events
MI: Myocardial infarction
MVO: Microvascular obstruction
NRI: Net reclassification improvement
PCI: Percutaneous coronary intervention
ROC: Receiver-operating characteristic
STEMI: ST-segment elevation myocardial infarction
T2WI: T2-weighted imaging
