# Supplementary material for: A contrast-enhanced CMR-based risk score integrating microvascular obstruction and intramyocardial hemorrhage extent for prognostic evaluation
Source: Insights Imaging. 2026 Aug 27;17:212. doi: 10.1186/s13244-026-02386-2 (PMC13522271; doi:10.1186/s13244-026-02386-2)
Supplement: Supplementary file 1 [file 13244_2026_2386_MOESM1_ESM.pdf]

A Contrast-Enhanced CMR-based Risk Score Integrating Microvascular Obstruction and Intramyocardial Hemorrhage Extent for Prognostic Evaluation

ELECTRONIC SUPPLEMENTARY MATERIAL

Insights Imaging (2026) Gu XY, Yin TX, Yu LY et al.

SUPPLEMENTAL MATERIAL

Manuscript title: A Contrast-Enhanced CMR-based Risk Score Integrating Microvascular Obstruction and Intramyocardial Hemorrhage Extent for Prognostic Evaluation

Supplemental Methods

Methods S1 Follow-Up and Clinical Outcome

The median follow-up duration after the initial STEMI event was 3.1 years (IQR: 1.7–5.0 years). Clinical information was collected from medical record reviews, telephone follow-ups, and outpatient visits. Patient outcomes were assessed at 6-month intervals.

The primary endpoint was major adverse cardiovascular events (MACE), defined as a composite of heart failure, all-cause death, reinfarction, and revascularization. Heart failure was strictly defined as an inpatient admission wherein heart failure constituted the principal diagnosis, mandating intravenous pharmacotherapy with diuretics, inotropic agents, or vasodilators. All-cause death included cardiovascular deaths attributable specifically to arrhythmia, myocardial infarction, heart failure, or sudden cardiac death, along with fatalities stemming from any other cause. Reinfarction refers to recurrent acute myocardial ischemic events occurring during follow-up after discharge for an initial myocardial

infarction, characterized by typical symptomatology and diagnosed according to the Fourth Universal Definition of Myocardial Infarction. Revascularization procedures specifically denoted non-staged percutaneous coronary interventions or coronary artery bypass grafts performed on the original culprit vessel during subsequent care following the index hospitalization.

Supplement Tables

Table.S1 Cardiovascular Magnetic Resonance Parameters.

Scanner	Philips			Siemens		
Sequence	Cardiac cine	LGE	T2WI-STIR	Cardiac cine	LGE	T2WI-STIR
Slice thickness (mm)	6	10	10	8	8	8
Flip angle (°)	45	25	90	46	10	160
TE (msec)	1.6	3	75	1.4	1.2	92
TR (msec)	3.2	6.1	/	35.42	742	/
Bandwidth, Hz/pixel	1800	256	600	960	790	445
Field of view (mm)	300*300	300*300	300*300	280*320	230*280	292*360

LGE: late gadolinium enhancement; T2WI-STIR (T2-weighted short-tau triple inversion recovery); TE: Echo Time (ms); TR: Repetition Time (ms).

Table.S2 Baseline characteristics of three groups divided by the existence of MVO and IMH among STEMI patients.

	MVO-/IMH- (n=299)	MVO+/IMH- (n=74)	MVO+/IMH+ (n=195)	P value
Age, years	62(55-68)	59(50-63)	61(53-67)	**0.003**
Men, n (%)	232(78)	66(89)	169(87)	**0.009**
BSA, m^2	1.8(1.7-1.9)	1.8(1.7-2)	1.9(1.7-1.9)	0.101
BMI, kg/m^2	24.7(22.3-27.0)	25.0(22.2-27.0)	24.6(23.0-26.9)	0.862
Heart rate, bpm	73(67-82)	76(70-85)	75(68-86)	0.100
GRACE score	86(76-104)	84(69-104)	95(76-114)	**0.005**
**Risk factors, n (%)**				
Hypertension	153(51)	39(53)	82(42)	0.100
Diabetes mellitus	109(36)	22(30)	88(45)	**0.038**
Dyslipidemia	88(29)	17(23)	41(21)	0.095
Smoking history	120(40)	53(72)	92(47)	**<0.001**
Killip class				**0.011**
Ⅰ	260(87)	59(80)	142(73)	
Ⅱ	31(10)	12(16)	42(22)	
Ⅲ	5(2)	1(1)	7(4)	
Ⅳ	3(1)	2(3)	4(2)	
**Laboratory indices**				
TnI_{max}, ng/ml	9.4(1.7-20.9)	11.5(4.4-24.5)	16.2(5.4-28.0)	**<0.001**
BNP_{max}, pg/ml	130(57-325)	264(74-562)	140(48-365)	**0.025**
NT-proBNP, pg/ml	1012(630-1510)	831(469-1484)	801(470-1330)	**0.045**
CRP_{max}, mg/l	4.3(1.2-29.6)	12.6(0.6-36.5)	1.6(0.5-8.3)	**<0.001**
hsCRP, mg/l	4.5(1.4-10.5)	3.4(1.7-7.3)	7.0(2.1-15.3)	**<0.001**
ApoA, mg/dL	102(49-171)	78(14-132)	118(58-204)	**0.001**
TC, mg/dL	4.4(3.7-5.3)	4.5(3.8-5.4)	4.5(3.6-5.4)	0.922
TG, mg/dL	1.5(1.0-2.4)	1.5(0.9-2.4)	1.5(1.0-2.3)	0.542
LDL, mmol/L	2.7(2.1-3.3)	2.8(2.1-3.4)	2.8(2.3-3.6)	0.164
HDL, mmol/L	1.0(0.9-1.3)	1.0(0.8-1.2)	1.0(0.9-1.2)	0.455
**Angiographic**				
Infarct artery				0.059
LAD	150(50)	37(50)	123(63)	
LCX	35(12)	9(12)	20(10)	
RCA	114(38)	28(38)	52(27)	
Number of diseased vessels				**0.046**
1	215(72)	56(76)	162(83)	
2	54(18)	9(12)	22(11)	

3	30(10)	9(12)	11(6)	
TIMI flow grade before PCI				**0.012**
0	191(64)	56(76)	140(72)	
1	8(3)	4(5)	12(6)	
2	23(8)	3(4)	16(8)	
3	77(26)	11(15)	27(14)	
TIMI flow grade after PCI				**0.022**
0	9(3)	5(7)	0(0)	
1	1(0)	0(0)	1(1)	
2	0(0)	1(1)	2(1)	
3	289(97)	68(92)	191(98)	
**Medications, n (%)**				
Aspirin	209(70)	43(58)	99(51)	**<0.001**
Other oral anti-platelet agents	206(69)	44(59)	101(52)	**0.001**
Statin	200(67)	46(62)	101(52)	**0.004**
ACE-I/ARB	112(37)	37(50)	54(28)	**0.002**
β-Blockers	171(57)	41(55)	94(48)	0.158
Diuretic	40(13)	11(15)	27(14)	0.943
Insulin/oral hypoglycemic agent	43(14)	19(26)	18(9)	0.003

Data are presented as median (interquartile range) or mean ± standard deviation or n/N (%). Values in **bold** indicate a P value <0.05. MACE: Major Adverse Cardiovascular Events; STEMI: ST-segment elevation myocardial infarction; BSA: Body Surface Area; BMI: Body Mass Index; GRACE: Global Registry of Acute Coronary Events; $cTnI_{max}$:peak troponin I; BNP_{max}: peak brain natriuretic peptide ; CRP_{max}: peak c-creative protein; TC: Total Cholesterol; TG: Triglycerides; LDL: low-density lipoprotein; HDL: high-density lipoprotein; LAD: left anterior descending; LCX: left circumflex; RCA: right coronary artery ; TIMI: Thrombolysis in Myocardial Infarction; ACEI: angiotensin converting enzyme inhibitor; ARB: Angiotonin Receptor Blocker.

Table.S3 CMR Parameters of three groups divided by the existence of MVO and IMH among STEMI patients.

Parameters	MVO-/IMH- (n=299)	MVO+/IMH- (n=74)	MVO+/IMH+ (n=195)	P value
LV EDVI (mL/m^2)	69.9(56.3-81.3)	76.4(67.5-94.3)	77.9(65.7-89.0)	**<0.001**
LV ESVI (mL/m^2)	30.1(23.2-41.2)	39.0(29.4-58.0)	38.9(31.4-50.7)	**<0.001**
LVEF (%)	51(42-58)	46(31-51)	47(38-53)	**<0.001**
LV SVi (mL/m^2)	36.0(26.9-43.0)	36.1(26.4-44.3)	36.6(30.1-43.8)	0.462
LV mass index (g/m^2)	58.4(47.3-73.3)	65.8(54.6-84.9)	64.6(55.7-74.2)	**<0.001**
LV aneurysm, n (%)	33(11)	25(34)	38(19)	**<0.001**
LV thrombus, n (%)	13(4)	11(15)	24(12)	**0.001**
LGE, % of LV	16(9-27)	20(17-30)	26(18-36)	**<0.001**

Data are presented as median (interquartile range) or mean ± standard deviation or n/N (%). Values in **bold** indicate a P value <0.05. CMR=Cardiac magnetic resonance; MVO: microvascular obstruction; IMH: intramyocardial hemorrhage; STEMI: ST-segment elevation myocardial infarction; LV: left ventricle; EDVI: end-diastolic volume index; ESVI: end-systolic volume index; LVEF: left ventricular ejection fraction; SVi = stroke volume index; LGE: late gadolinium enhancement.

Table S4. The distribution differences of CMR parameters categorized by the MVO and IMH cutoff value.

	MVO>1.06 (n=144)	MVO≤1.06 (n=424)	*P* Value	IMH>0.47 (n=129)	IMH≤0.47 (n=439)	*P* Value
MACE,n(%)	70(49)	38(9)	**<0.001**	63(49)	45(10)	**<0.001**
Survival time	684(367-1353)	1264(768-1929)	**<0.001**	842(373-1349)	1247(715-1939)	**<0.001**
LV EDVI (mL/m^2)	79.5(67.5-91.9)	71.0(59.0-84.4)	**<0.001**	78.6(67.2-90.5)	71.8(59.1-84.6)	**<0.001**
LV ESVI (mL/m^2)	41.2(32.9-56.8)	32.4(24.1-43.0)	**<0.001**	40.0(32.9-53.8)	32.8(24.0-44.0)	**<0.001**
LVEF (%)	44(35-50)	50(41-57)	**<0.001**	44(38-51)	49(41-57)	**<0.001**
LV SVi (mL/m^2)	36.1(27.8-43.3)	36.1(27.8-43.5)	0.994	36.0(28.4-42.5)	36.1(27.4-43.6)	0.886
LV mass index (g/m^2)	64.0(54.7-75.6)	60.5(49.3-75.4)	**0.027**	65.1(55.5-75.8)	60.5(49.1-75.4)	**0.018**
LV aneurysm, n(%)	34(24)	62(15)	**0.013**	28(22)	68(15)	0.098
LV thrombus,n (%)	16(11)	32(8)	0.184	17(13)	31(7)	**0.028**
LGE, % of LV	28(20-37)	18(10-28)	**<0.001**	29(22-38)	18(10-28)	**<0.001**

Data are presented as median (interquartile range) or mean ± standard deviation or n/N (%). Values in **bold** indicate a P value <0.05. CMR: Cardiac magnetic resonance; MVO: microvascular obstruction; IMH: intramyocardial hemorrhage; MACE: Major Adverse Cardiovascular Events; LV: left ventricle; EDVI: end-diastolic volume index; ESVI: end-systolic volume index; LVEF: left ventricular ejection fraction; SVi = stroke volume index; LGE: late gadolinium enhancement.

Table S5. The incidence of MACE events based on MVO & IMH Mass and CCS Stages.

	MVO≤1.06 IMH≤0.47 (n=398)	MVO≤1.06 IMH>0.47 (n=26)	MVO>1.06 IMH≤0.47 (n=41)	MVO>1.06 IMH>0.47 (n=103)	All cases (n=568)
CCS 2 (n=299)	10.4(31/299)	/	/	/	10.4%(31/299)
CCS 3 (n=74)	4.7%(2/43)	/	16.1%(5/31)	/	9.5%(7/74)
CCS 4 (n=195)	3.6%(2/56)	11.5%(3/26)	50%(5/10)	58.3%(60/103)	35.9%(70/195)
All cases (n=568)	8.8%(35/398)	11.5%(3/26)	24.4%(10/41)	58.3%(60/103)	19.0%(108/568)

The cutoff values for MVO and IMH are expressed as a percentage of left ventricular mass. MACE: Major Adverse Cardiovascular Events; MVO: microvascular obstruction; IMH: intramyocardial hemorrhage; CCS: Canadian Cardiovascular Society stages of acute myocardial infarction.

Table S6. Models Associated with CMR Parameters for Improving Predictive Ability of Specific Outcomes in STEMI Patients Based on AUC.

	Heart Failure		All Cause Death		Reinfarction		Revascularization	
	AUC	P value	AUC	P value	AUC	P value	AUC	P value
Grace Score	0.748	Reference	0.748	Reference	0.748	Reference	0.748	Reference
Score 1	0.822	**0.003**	0.790	**<0.001**	0.824	**<0.001**	0.798	**0.042**
Score 2	0.793	0.077	0.772	**0.001**	0.800	**0.010**	0.764	0.190
Score 3	0.843	**<0.001**	0.890	**<0.001**	0.850	**<0.001**	0.847	**<0.001**

Score 1 (GRACE Score, LVEF ≤47%, MI Size ≥19% LV and MVO >1.4% LV).

Score 2 (GRACE Score, LVEF ≤45% and IMH).

Score 3 (GRACE Score, LVEF ≤45%，MVO＞1.06%LV and IMH>0.47%LV).

CMR: Cardiac magnetic resonance; STEMI: ST-segment elevation myocardial infarction; AUC: area under the curve; GRACE: Global Registry of Acute Coronary Events; LVEF: left ventricular ejection fraction; MI: myocardial infarction; LV: left ventricle; MVO: microvascular obstruction; IMH: intramyocardial hemorrhage.

Table S7. Internal Validation of Risk Models Using Bootstrap Resampling (B=1000).

Model	Apparent AUC	Corrected AUC	95% CI	Optimism
Grace Score	0.748	0.750	0.698-0.799	-0.001
Score 1	0.825	0.819	0.784-0.866	0.006
Score 2	0.802	0.798	0.758-0.846	0.004
Score 3	0.853	0.849	0.813-0.892	0.004

Score 1 (GRACE Score, LVEF ≤47%, MI Size ≥19% LV and MVO >1.4% LV).

Score 2 (GRACE Score, LVEF ≤45% and IMH).

Score 3 (GRACE Score, LVEF ≤45%， MVO＞1.06%LV and IMH>0.47%LV).

AUC: area under the curve; CI: confidence interval; GRACE: Global Registry of Acute Coronary Events; LVEF: left ventricular ejection fraction; MI: myocardial infarction; LV: left ventricle; MVO: microvascular obstruction; IMH: intramyocardial hemorrhage.

Table S8. Intra- and inter-observer reproducibility of CMR parameters in patients with STEMI.

	Intra-observer		Inter-observer	
	ICC (95%CI)	P value	ICC (95%CI)	P value
LV EDVI (mL/m^2)	0.971(0.950-0.984)	**<0.001**	0.984(0.972-0.991)	**<0.001**
LV ESVI (mL/m^2)	0.962(0.935-0.978)	**<0.001**	0.992(0.986-0.996)	**<0.001**
LVEF (%)	0.966(0.928-0.984)	**<0.001**	0.990(0.978-0.995)	**<0.001**
LV SVi (mL/m^2)	0.928(0.850-0.966)	**<0.001**	0.986(0.970-0.994)	**<0.001**
LV mass index (g/m^2)	0.984(0.972-0.991)	**<0.001**	0.992(0.986-0.996)	**<0.001**
LGE, % of LV	0.973(0.954-0.985)	**<0.001**	0.991(0.984-0.995)	**<0.001**
MVO, % of LV mass	0.977(0.960-0.987)	**<0.001**	0.985(0.973-0.991)	**<0.001**
IMH, % of LV mass	0.961(0.933-0.977)	**<0.001**	0.962(0.934-0.978)	**<0.001**

CMR: Cardiac magnetic resonance; STEMI: ST-segment elevation myocardial infarction; ICC: interclass correlation coefficient; CI: confidence interval; LV: left ventricle; EDVI: end-diastolic volume index; ESVI: end-systolic volume index; LVEF: left ventricular ejection fraction; SVi = stroke volume index; LGE: late gadolinium enhancement; MVO: microvascular obstruction; IMH: intramyocardial hemorrhage.
